# The molecular and physiological roles of ABCC6: more than meets the eye

**DOI:** 10.3389/fgene.2012.00289

**Published:** 2012-12-12

**Authors:** Olivier Le Saux, Ludovic Martin, Zouhair Aherrahrou, Georges Leftheriotis, András Váradi, Christopher N. Brampton

**Affiliations:** ^1^Department of Cell and Molecular Biology, John A. Burns School of Medicine, University of HawaiiHonolulu, HI, USA; ^2^L'UNAM University, BNMI (UMR CNRS 6214/INSERM 1083), Angers School of MedicineAngers, France; ^3^Universitaet zu Luebeck, Institut Fuer Integrative und Experimentelle GenomikLuebeck, Germany; ^4^Institute of Enzymology, RCNS, Hungarian Academy of SciencesBudapest, Hungary

**Keywords:** pseudoxanthoma elasticum, ABCC6, calcification, β-thalassemia, generalized arterial calcification of infancy, ectopic cardiac calcification

## Abstract

Abnormal mineralization occurs in the context of several common conditions, including advanced age, diabetes, hypercholesterolemia, chronic renal failure, and certain genetic conditions. Metabolic, mechanical, infectious, and inflammatory injuries promote ectopic mineralization through overlapping yet distinct molecular mechanisms of initiation and progression. The ABCC6 protein is an ATP-dependent transporter primarily found in the plasma membrane of hepatocytes. ABCC6 exports unknown substrates from the liver presumably for systemic circulation. ABCC6 deficiency is the primary cause for chronic and acute forms of ectopic mineralization described in diseases such as pseudoxanthoma elasticum (PXE), β-thalassemia, and generalized arterial calcification of infancy (GACI) in humans and dystrophic cardiac calcification (DCC) in mice. These pathologies are characterized by mineralization of cardiovascular, ocular, and dermal tissues. PXE and to an extent GACI are caused by inactivating *ABCC6* mutations, whereas the mineralization associated with β-thalassemia patients derives from a liver-specific change in ABCC6 expression. DCC is an acquired phenotype resulting from cardiovascular insults (ischemic injury or hyperlipidemia) and secondary to ABCC6 insufficiency. *Abcc6*-deficient mice develop ectopic calcifications similar to both the human PXE and mouse DCC phenotypes. The precise molecular and cellular mechanism linking deficient hepatic ABCC6 function to distal ectopic mineral deposition is not understood and has captured the attention of many research groups. Our previously published work along with that of others show that ABCC6 influences other modulators of calcification and that it plays a much greater physiological role than originally thought.

## Introduction

In the absence of any systemic mineral imbalance, the calcification of soft tissues is defined as ectopic or dystrophic calcification. The latter term specifically distinguishes ectopic calcification that occurs in injured, damaged, and/or necrotic tissues. In contrast, elevated levels of calcium and/or phosphate due to abnormal absorption and/or secretion lead to metastatic mineralization. Whether ectopic or metastatic, abnormal calcification is typically composed of calcium phosphate salts, such as hydroxyapatite and can affect most soft tissues. However, the skin, kidneys, tendons, and cardiovascular tissues are particularly prone to this pathology.

Vascular calcification is a prevalent feature of aging and is also frequently associated with a number of common pathologies that include hyperlipidemia (atherosclerosis), chronic renal insufficiency, and diabetes, as well as certain infrequent genetic conditions. Calcification was long thought to result from passive precipitation of calcium and phosphate but it is now recognized as a complex tightly regulated development involving the osteoblastic differentiation of resident cells such as smooth muscle cells (SMCs), pericytes or adventitial myofibroblasts. It was also viewed as harmless, but calcification is in fact causative in the precipitation of cardiovascular events and mediating chronic damages to these tissues independently of the disease context that brings it about. More importantly, a proliferation of recent data has brought into light the many factors and the complex mechanisms that initiate and promote calcification *in vivo* (Atzeni et al., 2006). Despite the significant improvement of our understanding of calcification processes, how the delicate balance between normal osteogenic signals and ectopic mineralization in soft tissues is altered in pathological conditions is far from being understood.

Based on multiple evidence gathered in the last decade, the ATP-binding cassette (ABC) transporter ABCC6 has joined the list of calcification regulators as a new member. Indeed, reduced levels of ABCC6 protein or loss of ABCC6 function in the liver has been linked to four separate ectopic mineralization pathologies in humans and mice. (1) Pseudoxanthoma elasticum (PXE: MIM 264800) is an autosomal recessive disease characterized by a slow and progressive ectopic calcification primarily affecting elastic fibers in dermal, ocular, and vascular tissues. Inactivating mutations in the *ABCC*6 gene cause PXE (Bergen et al., 2000; Le Saux et al., 2000, 2001). (2) We have also shown, based on results obtained with an animal model, that the calcification phenotype in some β-thalassemia patients of Mediterranean descent (Baccarani-Contri et al., 2001; Aessopos et al., 2002), while not directly caused by *ABCC*6 gene mutations (Hamlin et al., 2003), probably results from reduced levels of ABCC6 protein in the liver. (3) Furthermore, generalized arterial calcification of infancy (GACI) is another heritable disorder typically associated with mutations in the *ENPP1* gene. It now appears that a significant fraction of patients diagnosed with GACI are in fact carriers of *ABCC*6 mutations while typical PXE manifestations can be associated with ENPP1 mutations in some young patients (Le Boulanger et al., 2010; Nitschke et al., 2012). (4) Recently, two groups of investigators have established that deficiency of the ABCC6 protein is linked to an acute calcification phenotype affecting the myocardium and the media of large arteries in several inbred strains of mice, including C3H/HeJ and DBA/2J (Aherrahrou et al., 2004, 2008; Meng et al., 2007). Because this peculiar phenotype occurs in response to a tissue injury it is referred to as dystrophic cardiac calcification or DCC.

The aim of this review is to summarize present knowledge on ABCC6 function and its possible molecular and physiological roles in various calcification pathologies where there clearly is more than meets the eye with this unique ABC transporter.

## The molecular characteristics of ABCC6

### ABCC6 is an efflux pump

ABCC6 is a member of the large ABC gene subfamily C. In this group of transmembrane proteins, in addition to active transporters such as ABCC1, −2, −3, −4, and −5, there are also ion channel-forming proteins and ion channel regulators like ABCC7 (CFTR), ABCC8 and −9 (SUR1 and −2). The work of Ilias et al. (2002) and Belinsky et al. (2002) have shown that ABCC6 is a genuine active efflux transporter that uses ATP to effectively pump a glutathione conjugate of N-ethylmaleimide (NEM-GS) and also leukotriene C4 (LTC4). The affinity of ABCC6 for LTC4 was much lower than for NEM-GS and overall the maximal rate of NEM-GS transport was markedly inferior to two other well-known glutathione conjugate-transporting proteins, ABCC1 and ABCC2. Interestingly, ABCC6 failed to effectively transport 17-β-estradiol-17-β-D-glucuronide that is otherwise a recognized a substrate for ABCC3 (Hirohashi et al., 1999). The same study also found that the most effective inhibitors for ABCC6 were benzbromarone and indomethacin. These *in vitro* observations demonstrated that ABCC6 has a defined perhaps restricted, substrate specificity. Though the actual endogenous substrate(s) for this transporter and hence the contributing factor to ectopic calcification in PXE, β-thalassemia, GACI, and DCC remains unknown.

### ABCC6 structure

A 3D configuration of ABCC6 was successfully modeled using the X-ray structure of the *Staphylococcus aureus* Sav1866 export pump (Dawson and Locher, 2007). This prokaryote pump has already been used as template to build other homology models for the several human ABCC transporters such as ABCC1 (DeGorter et al., 2008), ABCC4 (Ravna and Sager, 2008), ABCC5 (Ravna et al., 2007), ABCC7/CFTR (Serohijos et al., 2008) as well as ABCB1 (Zolnerciks et al., 2007), ABCG2 (Li et al., 2007). Fülöp et al. have used their model of ABCC6 and the distribution of PXE-causing mutations to demonstrate the strict relevance of the transmission interface (ICL-ABC contacts) as well as the ABC-ABC domain contacts for the function of the transporter (Fulop et al., 2009). For more information on the structure/function relation of ABCC6, see the review of Arányi et al. in this issue.

### Cellular localization of ABCC6

ABCC6 protein has thus far been unambiguously associated with the basolateral membrane of hepatocytes in mice, rat, and human samples (Madon et al., 2000; Beck et al., 2003; Le Saux et al., 2011) as well as in the proximal kidney tubules and a kidney cell line (Sinko et al., 2003; Beck et al., 2005). Although a recent publication is unconvincingly attempting to challenge the already well-defined cellular localization of ABCC6 (Martin et al., 2012), it is interesting to note that PXE-causing missense mutations in ABCC6 do lead to defective cellular localization of the protein along with other functional alterations of the translated proteins (Le Saux et al., 2011). Although a relatively small number of mutants were analyzed, two possible outcomes of pathological mutations were described: (1) failure to use ATP causing transport deficiency and (2) the altered folding and/or protein stability leading to intracellular retention and reduced trafficking or a combination thereof. Therefore, the various structural and functional alterations of mutated ABCC6 presumably all result in the loss of physiological function, which provides a reasonable explanation for the observed lack of phenotype–genotype correlation in PXE (Le Saux et al., 2001; Chassaing et al., 2005; Pfendner et al., 2007).

### ABCC6 substrate(s) conundrum

**Figure 1:**
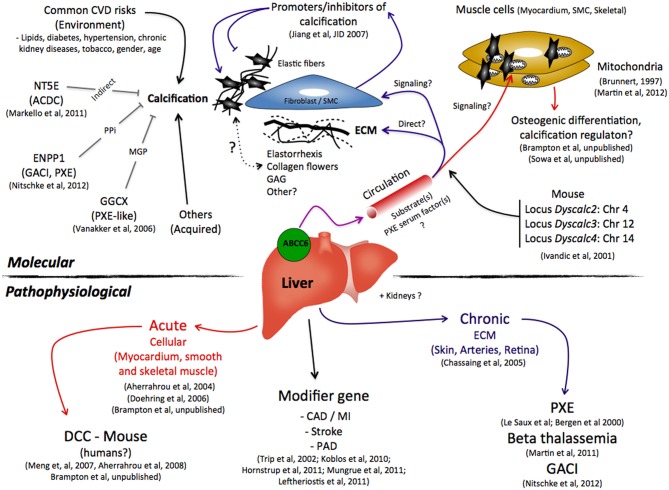
**A summary of the possible molecular and pathophysiological roles that ABCC6 plays in the ectopic calcification as seen in pseudoxanthoma elasticum, β-thalassemia, generalized arterial calcification of infancy, dystrophic cardiac calcification, and other cardiovascular diseases**.
Abbreviations:CADCoronary Artery DiseasesCVDCardiovascular DiseasesECMextracellular matrixGAGGlycoaminoglycansMGPMatrix Gla ProteinMIMyocardial InfarctionPADPeripheral Arterial DiseasePPipyrophosphateSMCSmooth Muscle Cells.

Since the identification of *ABCC*6 as the causative gene for PXE (Bergen et al., 2000; Le Saux et al., 2000), the question of its substrate(s) has thus far eluded all interested parties, and indeed the identification of an endogenous substrate(s) for an ABC transporter is not an easy task. Our knowledge to date is based on limited experimental data showing ABCC6's ability to use ATP to extrude conjugated metabolites *in vitro* (Belinsky et al., 2002; Ilias et al., 2002). Because the protein rests in the basolateral membrane of polarized cells, the prevailing hypothesis stipulates that the inability of this transporter to secrete its unknown substrate(s) for systemic circulation is the primary cause of the ectopic calcification phenotype of PXE, some GACI, and β-thalassemia patients and for DCC. This has prompted some to describe PXE as a metabolic disorder (Uitto et al., 2001), which could also apply to DCC and the ABCC6-dependent GACI and β-thalassemia cases. The metabolic hypothesis implies that the ABCC6 substrate(s) ultimately acts as an inhibitor of calcification in peripheral tissues. Does it work as a signaling molecule(s) that diffuses from the circulation into connective tissues where it contributes the normal phenotype of resident cells such as fibroblasts, or SMCs (Quaglino et al., 2000; Boraldi et al., 2003)? Or does it interact directly with the extracellular matrix, collagen fibrils, elastic fibers thereby precluding oxidative stress (Garcia-Fernandez et al., 2008), abnormal assembly/deposition of collagen fibrils (Gheduzzi et al., 2003) and elastic fiber (Le Saux et al., 2006) calcification and fragmentation? As multiple structural and molecular alterations have been noted in PXE notably the so-called “collagen flowers,” elastorrhexis along with abnormal glycoaminoglycans depositions (Lebwohl et al., 1993; Passi et al., 1996; Quaglino et al., 2000; Baccarani-Contri et al., 2001; Gheduzzi et al., 2003; Gotting et al., 2005) it is possible that a combination of molecular and cellular processes contribute to all the molecular and structural changes that have been described up to now (Figure 1). These interrogations clearly reflect a lack of sufficient data to even speculate as to the nature of the ABCC6 physiological substrate(s). And, in such situations, there are two possible approaches. Either one tests candidate molecules or performs a systematic search.

### Candidate ABCC6 substrates

Reports by Vanakker and co-workers prompted several laboratories to test one such candidate molecule. The first of two publications described a PXE-like syndrome caused by a deficiency in gamma-glutamyl carboxylase (GGCX) (Vanakker et al., 2007) and a second article reported a deficit in the carboxylation (activation) of Gla proteins, including the calcification inhibitor Matrix Gla Protein (MGP), secondary to reduced vitamin K levels in the circulation of PXE patients (Vanakker et al., 2010). Because the common denominator between both reports is the vitamin K, the speculation that this co-factor could be an ABCC6 substrate was proposed (Borst et al., 2008) and tested in both indirect and direct experiments. The indirect approaches focused on phenotype correction in animal models of PXE, namely the calcification of vibrissae, with diets enriched in phylloquinone (vitamin K_1_) and menaquinones (vitamin K_2_). Three independent studies were carried out and all obtained negative results (Brampton et al., 2011; Gorgels et al., 2011; Jiang et al., 2011). These results were later confirmed by the direct observation that ABCC6 failed to effectively transport a glutathione conjugate of vitamin K_3_, unlike ABCC1 (Fulop et al., 2011). Vitamin K_3_ is an intermediate generated during the conversion of the dietary vitamin K_1_ to the most abundant form vitamin K_2_.

More recently, another candidate molecule was tested on the premise that adenosine has a key role in the etiology of arterial calcification due to deficiency of CD73 (ACDC), a rare condition that has phenotypic similarities with PXE (Markello et al., 2011). The ACDC mineralization primarily affects the large lower limb arteries (femoral, popliteal, and tibial arteries) and cartilage tissues but spares the main arterial vessels of the upper body. ACDC results from mutations in the *NT5E* gene that encodes the 5′ exonucleotidase CD73. This protein is a glycosyl phosphatidylinositol-anchored plasma membrane protein that generates extracellular adenosine, downstream of ENPP1 as part of the extracellular degradation pathway from ATP to adenosine and inorganic phosphate. The calcification in ACDC patients derives from an increase tissue non-specific alkaline phosphatase (TNAP) expression as a consequence of the lack of adenosine signaling. The partial overlap between the PXE and ACDC phenotypes prompted Markello et al. to suggest that adenosine might be an ABCC6 substrate (Markello et al., 2011). However, the limitations of this theory were quickly apparent as CD73 contributed to calcification only in specific arterial territories without dermal or ocular involvement (Leftheriotis et al., 2011b). Subsequent *in vitro* testing showed that indeed ABCC6 did not efficiently transport adenosine (Szabo et al., 2011).

### The systematic search for ABCC6 substrate(s)

As for systematic approaches, at present three groups of laboratories are reportedly undertaking experiments using animal or human tissue extracts and fluids that potentially contain the ABCC6 substrate(s). Though no publications or public reports has been made thus far, two of these groups have based their technical approaches on the principle described by Krumpochova et al. (2012) which use of a combination of inverted membrane vesicles from *Spodoptera frugiperda* (Sf9) insect cells overproducing ABCC6 and liquid chromatography/mass spectroscopy-based metabolomics to determine the compounds transported into the vesicles (Personal communications, O. Le Saux, A. Varadi, and P. Borst). The third group of laboratories actively seeking the ABCC6 substrate(s) uses the methodology reported by Van et al. (2008): NMR spectroscopy with animal and/or human urine and serum (Personal communications M. Dean).

### The tip of the iceberg

As of today, the sum of current publications only paints a relatively fragmented picture of the pathophysiological role of ABCC6 that seem to favor a signaling/hormone-type of activity for ABCC6 substrate(s) toward a variety of tissues, including but not limited to, the connective tissue. For example, the observations that Fetuin-A, MGP (Hendig et al., 2006, 2008b), vitamin K (Vanakker et al., 2010), osteogenic makers, and oxidative stress (Pasquali-Ronchetti et al., 2006; Garcia-Fernandez et al., 2008; Hendig et al., 2008a) are altered in the circulation of PXE patients points indirectly to unbalanced homeostasis of multiple organs and tissues. More direct evidence have shown the presence of pathological metabolite(s) in the serum of PXE patients and *Abcc*6^−/−^ mice that promoted elastic fibers structural alterations and ectopic calcification (Le Saux et al., 2006; Jiang et al., 2007). Further, we have also described a significant expansion of the lymphatic vessel network in *Abcc*6^−/−^ mice (Le Corre et al., 2012) that emphasize the systemic and global nature of the physiological changes that accompany and/or lead to the mineralization phenotype linked to ABCC6 deficiency. This calcification has always been presented as the hallmark of PXE and the major pathological development DCC and GACI, but it might in fact be only the visible tip of the iceberg.

## The pathophysiologies associated with ABCC6 deficiency

Little is known about ABCC6 other than it is a member of the large ABC gene subfamily C (multi drug resistance proteins) and encodes a transmembrane protein that uses ATP hydrolysis to export organic anion transport across cellular membranes. Work to characterize the physiological role of ABCC6 has first relied upon tissue expression profiles. ABCC6 is found primarily expressed in liver and the kidneys but is also in the intestine, the retina and to a much lesser extent in most other tissues including the lung, skin, and vasculature (Bergen et al., 2000; Beck et al., 2003, 2005). Although it is unclear if ABCC6 has a unique function distributed in multiple tissues or tissue-specific roles, its deficiency was first and foremost linked to calcification activation in PXE, β-thalassemia, and GACI in humans and DCC in mice. As off today, only two studies point to the liver as the tissue with the most relevance with respect to the calcification phenotype (Martin et al., 2011; Brampton et al., 2012).

### Pseudoxanthoma elasticum

PXE (MIM 264800) is a recessive disease affecting the connective tissue and is defined by the calcification and fragmentation of elastic fibers (Chassaing et al., 2005). The PXE clinical signs primarily involve the skin, the Bruch's membrane of the eyes and cardiovascular system resulting in skin sagging and redundancy, visual impairment caused by retinal hemorrhages and peripheral arterial disease (PAD) associated with gastrointestinal bleeding and intermittent claudication. The localization of the transmembrane ABCC6 protein into the basolateral plasma membrane of hepatocytes and proximal kidney tubules cells is very relevant to its physiological function. It is its inability to secrete its unknown substrate toward the circulation that is the most likely cause of the ectopic calcification in PXE, which describes this disease as metabolic rather than a connective tissue disease.

### β-thalassemia

The second calcification phenotype linked to ABCC6 is in fact a phenocopy of PXE. β-thalassemia (MIM 141900) is a monogenic disorder caused by mutations in the β-globin gene that leads to the underproduction of β-globin chains. The stoichiometric excess of α-chains unbound to β-globin is unstable and precipitate in red blood cell precursors forming inclusion bodies. These are responsible for the intramedullary destruction of the erythroid precursors and the ineffective erythropoiesis that characterize the β-thalassemias. Ineffective erythropoeisis in thalassemia major and certain intermedia patients results in considerable marrow expansion causing bone deformities and iron overload that is further exacerbated by frequent blood transfusions (Thein, 1998). The β-thalassemias are widespread throughout the Mediterranean, Africa, the Middle East, the Indian subcontinent, and Southeast Asia. In the past decade, it has become apparent that a large number of Mediterranean patients affected by β-thalassemia or sickle cell anemia also develop manifestations similar to PXE (Aessopos et al., 2002). β-thalassemia and PXE are distinct genetic disorders yet, the ectopic mineralization phenotype of seen in β-thalassemia patients is clinically and structurally identical to inherited PXE (Baccarani-Contri et al., 2001; Cianciulli et al., 2002; Farmakis et al., 2003, 2004). As we have established that the PXE-like mineralization in β-thalassemia patients arises independently of *ABCC*6 mutations (Hamlin et al., 2003), we hypothesized that the expression of the *ABCC*6 gene or the biological properties of its product could be disrupted in liver and/or kidneys as a secondary consequence of the hemoglobinopathy. We have tested this possibility by following the synthesis of ABCC6 in the liver and kidneys of a β-thalassemia mouse model (*Hbb*^*th3/+*^). We found a progressive liver-specific downregulation in the *Abcc*6 gene expression and the corresponding protein levels. This downregulation became significant at 6 months of age and stabilized at older ages at ~25% of the wild type protein levels. Studying the transcriptional regulation of the *Abcc*6 gene revealed that the main cause of the downregulation resided with the absence of a single transcription factor, the erythroid-specific NF-E2 from the *Abcc*6 promoter. Coincidentally, NF-E2 is a major transcription factor for the expression of several hemoglobin-related genes (Andrews, 1998). *Hbb*^*th3/+*^mice did not develop spontaneous calcification as seen in *Abcc*6^−/−^ mice probably because the ABCC6 protein decrease occurred late in life and/or was insufficient to promote mineralization in the *Hbb*^*th3/+*^ mouse with the DCC-resistant C57BL/6J genetic background (Martin et al., 2011). Nevertheless, as the transcriptional regulation of the mouse and human *ABCC*6 genes is similar (Aranyi et al., 2005; Douet et al., 2006, 2007; de Boussac et al., 2010; Ratajewski et al., 2012), it is likely that the human β-thalassemia phenotype could induce comparable molecular changes leading to a suboptimal endowment in ABCC6 and increased susceptibility to ectopic mineralization in a PXE-like manner.

### Generalized arterial calcification of infancy

**Figure 2:**
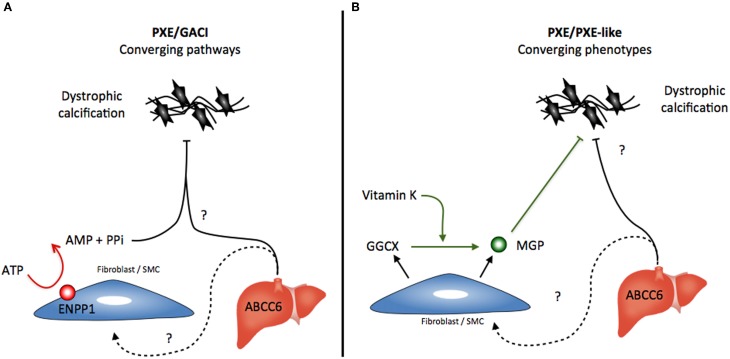
**Possible scenario for the converging (A) pathways that lead to the calcification phenotypes caused by ENPP1 and ABCC6 deficiencies and **(B)** the converging of the dissimilar PXE and GGCX-dependent PXE-like syndrome**.
Abbreviations:PPipyrophosphateMGPMatrix Gla Protein.

The third disease related to the ABCC6 deficiency is GACI, a rare autosomal-recessive disorder characterized by severe pathologic calcifications in the arterial media with intimal proliferation leading to vascular occlusion. GACI is associated with biallelic mutations in *ENPP1* and affected patients suffer from hypertension, severe myocardial ischemia and congestive heart failure. Most affected patients die within the first 6 months of life. The obvious overlapping mineralization phenotype between GACI and PXE led to a recent study that correlated the phenotype to genotype in GACI and PXE patients (Nitschke et al., 2012). This work found clinical manifestations unique to PXE in GACI patients carrying *ENPP1* mutations including angioid streaks and identical skin lesions. Additionally, mutations in *ABCC*6 accounted for a significant subset of GACI patients where no *ENPP1* mutation was found. The authors concluded that PXE and GACI are in fact diseases with overlapping characteristics reflecting a spectrum of severity in ectopic calcification rather than two distinct entities (Nitschke et al., 2012). However, the clear resemblances between the GACI and PXE phenotypes rather suggests an underlying convergence of ENPP1 and ABCC6 molecular pathways toward a common inhibition of mineralization somewhere upstream of the phenotypic manifestations, i.e., calcification because *ENPP1* deficiency leads to elastic fiber alterations typical of PXE in the vasculature, the skin, and ocular tissues (Figure 2A).

A somewhat similar convergence exists between the PXE-like syndrome in which *GGCX* mutations lead to insufficient MGP carboxylation (activation) and the classic inherited PXE (Gheduzzi et al., 2007; Vanakker et al., 2010). Unlike the PXE/GACI connection, the similarities between PXE and GGCX-associated syndrome represent a convergence of phenotypes rather than merging pathways (Figure 2B). The similarities are probably only due to the involvement of MGP deficiencies in both diseases. The patterns of elastic fiber mineralization are structurally different and the clinical evolution of the PXE-like syndrome diverges from PXE notably with a much greater laxity of the skin (Vanakker et al., 2007). A similar paradigm exists for other diseases such as *cutis laxa*, which can be either inherited through mutations in several genes related to elastic fibers or acquired through other processes affecting the structural integrity of these elastic fibers (Berk et al., 2012).

### The murine dystrophic cardiac calcification

#### DCC

In recent years, two groups of investigators have established that ABCC6 deficiency is linked to an acute dystrophic calcification phenotype affecting the myocardium of several inbred strains of mice, including C3H/HeJ, 129S1/SvJ, and DBA/2J (Doehring et al., 2006; Meng et al., 2007; Aherrahrou et al., 2008). This murine phenotype is designated DCC. It is an autosomal recessive trait that was described several decades ago in animal models (Eaton et al., 1978; Everitt et al., 1988). It corresponds to a condition affecting cardiac tissues that can either occur spontaneously over the long-term or be initiated by specific dietary regime. Importantly, DCC can develop into an acute phenotype if triggered by a severe injury including surface freeze-thaw injuries (Ivandic et al., 2001; Aherrahrou et al., 2004) or ischemia (Brampton et al., 2012). In addition to cardiac tissues, the vasculature, notably the aortic artery (SMCs) as well as skeletal muscles, are also susceptible to developing calcification in response to the same type of severe injuries (Brunnert, 1997; Doehring et al., 2006). The major locus controlling this trait was first mapped to chromosome 7 (Ivandic et al., 2001) and subsequently was linked to a single *Abcc*6 gene mutation in C3H/HeJ mice that leads to a large constitutive decrease in ABCC6 protein levels in the liver (Aherrahrou et al., 2008). The same mutation is present in 129S1/SvJ and DBA/2J mouse strains while it is absent in C57BL/6J mice that are DCC-resistant. There are three additional minor loci affecting the penetrance and the expression of the DCC phenotype that were mapped to chromosomes 4, 12, and 14 (Ivandic et al., 2001), though no specific genes have been identified as yet.

DCC-susceptible C3H/HeJ mice develop an attenuated version of the murine PXE phenotype as compared to the *Abcc*6^−/−^ animals, while the DBA/2J mice present little or no manifestations (Smolen et al., 2012). It is interesting to note that the murine PXE manifestations recently reported in KK/H1J mice are remarkably severe and somewhat more extended than those of the *Abcc*6^−/−^ mice (Li et al., 2012). More interesting is that all these strains of mice carry the exact same *Abcc*6 gene mutation, which clearly underlines the influence of the genetic background in dystrophic calcification and thus the synergistic convergence of multiple gene-encoded pathways toward a common end-result that is the pathological mineralization of soft tissues (Figures 1 and 2).

In a study that we have now submitted for publication, we specifically explored the role of ABCC6 in the calcification response to cardiovascular insults. We used two different models of infarction, the non-ischemic freeze-thaw (cryoinjury) and coronary artery ligation. We first confirmed the propensity to cardiac mineralization of *Abcc*6^−/−^ mice backcrossed into the DCC-resistant C57BL/6J background and thus the primordial role of ABCC6 in acute calcification. Furthermore, we have successfully modulated the calcification response to cryoinjury by varying the expression levels of ABCC6, either using heterozygous *Abcc6^+/−^* mice or the transient expression of the human ABCC6 protein in the liver of *Abcc6*-null mice. Moreover, the levels of *ABCC*6 correlated with the amount and distribution of the regulators of mineralization osteopontin (OPN) and MGP but not osteocalcin (OC) clearly indicating that ABCC6 regulates cardiac calcification in conjunction with the local regulators of mineralization (Brampton et al., 2012).

#### Mitochondrial calcification

In 1997, Brunnert reported for the first time the precipitation of amorphous calcium within and around swollen mitochondria, a few hours after myocardial damage in the DCC-susceptible C3H/HeJ and DBA/2J mice. Subsequently, these electron-dense calcium deposits grew larger encompassing the entire cytoplasm and eventually the surrounding myofibrils (Brunnert, 1997). These findings prompted the author to hypothesize that dystrophic calcification may actually result from altered mitochondrial function. A suggestion that the recent report from Martin et al. has partially corroborated by showing that mitochondria in cardiac, liver, and renal tissues of *Abcc*6^−/−^ mice were structurally altered and presented decreased respiratory capacities (Martin et al., 2012). Later, one of us (Aherrahrou, Z.) as well as others confirmed the formation of hydroxyapatite in cardiomyocytes mitochondria of C3H/HeJ mice (Aherrahrou, 2003). And interestingly, hydroxyapatite crystals in the mitochondria of cardiomyocytes were present equally in both C3H/HeJ mice and the DCC-resistant C57BL/6 animals in the first 24 h after cardiac injury. That changed in the following days when the growth of calcium crystal continued to spread in the C3H/HeJ mice while it subsided in C57BL/6J (Aherrahrou, 2003). These data suggested that in both strains, the mitochondria were first be able to sequester and concentrate calcium salts beyond solubility in the injured cells. And indeed, the key role that the ATP-dependent mitochondrial calcium sequestration exerts on intracellular calcium stores during cell death processes has largely been documented (Chakraborti et al., 1999; Raha and Robinson, 2001). However, the runaway formation of hydroxyapatite crystals in C3H/HeJ mice, which also requires the involvement of phosphate ions, appears to be linked to an ABCC6-dependent deficiency of a calcification inhibition from within the mitochondria. It is unclear what this calcification inhibition might be and whether the rapid progression of crystal formation in C3H/HeJ mice is connected to the abnormal respiration function of mitochondrial as noted by Martin et al. (2012). Though, one would wonder if vitamin K_2_, which has recently been described as an electron carrier in mitochondria (Vos et al., 2012), participates in this acute calcification phenotype especially in the light of the large discrepancy we previously described in the levels of circulating vitamin K_1_ and K_2_ between *Abcc*6^−/−^mice and C57BL/6J controls animals fed an enriched diet (Brampton et al., 2011).

#### DCC vs. PXE

One must distinguish the fundamental differences that exist between the induced calcification phenotype of DCC mice and the mineralization seen in the prototypic PXE disease. The latter phenotype is characterized by a long-term chronic and passive development of calcification that primarily affects the extracellular matrix (elastic fibers) over a period of time counted in years for humans and in months for mice. In contrast, the DCC phenotype is acute when induced and develops over a very short period of no more than 72 h, seemingly affecting only non-elastic muscular tissues. Moreover, the induced DCC calcification is intracellular, occurring within mitochondria. Both chronic and acute molecular pathways leading to calcification share the same molecular origin, i.e., ABCC6 deficiency. However, their mechanism of initiation and progression are clearly different which indicate that ABCC6 signaling (from the liver) has much broader ramifications toward a variety of cellular and molecular processes than we originally thought. Of note, the DCC phenotype has not (yet) been described in human PXE patients, though cardiac calcification occurring within cardiomyocytes mitochondria is not uncommon after severe myocardial damages following an ischemic event (Bloom and Peric-Golia, 1989; Lockard and Bloom, 1991).

### Vitamin K and MGP-dependent inhibition of calcification

As discussed above, vitamin K or one of its derivatives is not a substrate transported by ABCC6, though the depleted levels of circulating vitamin K in PXE patients was thought to have a direct consequence in the carboxylation (activation) status of the calcification inhibitor MGP and the susceptibility to chronic calcification in PXE (Gheduzzi et al., 2007; Vanakker et al., 2007, 2010). We have shown that increasing the availability of vitamin K_1_ or K_2_ in peripheral tissues of *Abcc*6^−/−^ mice did not significantly affect the MGP carboxylation status in the calcified capsule of vibrissae (Brampton et al., 2011). And going further, Boraldi et al. (2012) have now established that dermal fibroblasts isolated from PXE patients were able to uptake and use vitamin K_1_ or K_2_ for overall protein carboxylation as efficiently as healthy fibroblasts but not for MGP, which remained specifically undercarboxylated. As described above, we found that variable ABCC6 expression levels in the liver modulated the amounts of undercarboxylated MGP in calcified cardiac tissues (Brampton et al., 2012). Taken together these results suggested that MGP or the regulation of its carboxylation process and possibly OPN correlate with ABCC6 signaling and/or the ectopic calcification status.

### ABCC6 as a phenotype modifier gene

#### Infarct size

A recent study by Mungrue et al. (2011) suggested a relationship between ABCC6 function and infarct size under short-term ischemia reperfusion conditions (under an hour). In their studies, the authors noted the absence of any calcification in the myocardium of *Abcc*6-null mice suggesting that only a sustained cardiac injury lead to significant tissue necrosis and calcification in the absence of ABCC6 function (Figure 1).

#### Susceptibility to common artery diseases

The independent report by Köblös et al. as well as Trip and co-workers have both suggested that human heterozygous carriers of *ABCC*6 mutations are more likely to develop complications resulting from cardiovascular incidents than the general population (Trip et al., 2002; Koblos et al., 2010). However, this is not without controversy as a much larger study based on 66,831 individuals has found no risk for ischemic heart diseases associated with the ABCC6 p.R1141X mutation (Hornstrup et al., 2011). Stroke is also a vascular-related condition frequently reported in PXE patients (Aessopos et al., 1997; van den Berg et al., 2000) but it could well be that strokes etiology in certain PXE individuals might not be related to ABCC6 deficiency as Hornstrup et al. could not statistically link cerebrovascular diseases with the most frequent ABCC6 mutation (p.R1141X). The occurrence of PAD in PXE (Figure 1) is less contentious as its precise characteristics are being carefully studied in a French cohort (Leftheriotis et al., 2011a). For more details on the prevalence and the peculiar presentation of PAD in PXE, see the review of Leftheriotis et al. in this issue.

## Concluding remarks

For many years, *ABCC*6 was considered to have little more relevance than the causative gene for a rare heritable disease, PXE. However, we and others have now assembled a large body of data that clearly demonstrates that ABCC6 is far more important for cardiovascular health, in aging, and multiple diseased states than was initially thought. ABCC6 deficiency not only increases directly the susceptibility to connective tissue (elastic fibers) calcification in PXE, it also contributes to and aggravates the pathology of a significant fraction of GACI and β-thalassemia patients. This protein is the root-cause of an acute mineralization phenotype that when triggered dramatically affects the intracellular calcium homeostasis in muscle tissues. ABCC6 is now a fully fledged inhibitor of calcification that works at a systemic level (through the circulation) as part of a larger ensemble of local and general regulators of calcification. But, the loss of ABCC6 function also leads to various physiological changes other than calcification that we have only begun to describe and undoubtedly, more is yet to come.

### Conflict of interest statement

The authors declare that the research was conducted in the absence of any commercial or financial relationships that could be construed as a potential conflict of interest.
